# Shifting Carbon Fractions in Forest Soils Offset ^14^C‐Based Turnover Times Along a 1700 m Elevation Gradient

**DOI:** 10.1111/gcb.70326

**Published:** 2025-07-12

**Authors:** Margaux Moreno‐Duborgel, Sia Gosheva‐Oney, Beatriz González‐Domínguez, Mirjam Brühlmann, Luisa I. Minich, Negar Haghipour, Roman Flury, Claudia Guidi, Alexander S. Brunmayr, Samuel Abiven, Timothy I. Eglinton, Frank Hagedorn

**Affiliations:** ^1^ Swiss Federal Institute for Forest, Snow and Landscape Research (WSL) Zurich Switzerland; ^2^ Department of Earth and Planetary Sciences ETH Zurich Zurich Switzerland; ^3^ Department of Evolutionary Biology and Environmental Studies University of Zurich (UZH) Zurich Switzerland; ^4^ Department of Geography, Soil Science and Biogeochemistry Unit University of Zurich (UZH) Zurich Switzerland; ^5^ Laboratory for Ion Beam Physics, Department of Physics ETH Zurich Zurich Switzerland; ^6^ Department of Physics Imperial College London London UK; ^7^ Laboratoire de Géologie CNRS—École Normale supérieure, PSL University Paris France; ^8^ Centre de Recherche en Ecologie Expérimentale et Prédictive (CEREEP‐Ecotron Ile de France) Ecole Normale Supérieure, CNRS, PSL Research University Paris France

**Keywords:** C stocks, climate, elevation gradient, mineral‐associated organic matter (MOM), particulate organic matter (POM), radiocarbon (^14^C), soil organic matter fractions, turnover time, δ^13^C and δ ^15^N

## Abstract

Climate change impacts the soil carbon cycle, yet there is no scientific consensus on the vulnerability of soil organic carbon (SOC) stocks to global warming. Here, we studied soil organic matter (SOM) changes across 50 Swiss forest sites covering an elevation gradient from 270 to 2020 m, with dominant tree species changing from sub‐Mediterranean pubescent oak to mountain pine at treeline. We sought to assess how elevation, serving as an integrating variable for climate variation, affects the stocks, transformation state, and radiocarbon (^14^C)‐based turnover of SOM fractions in the organic layer, as well as in particulate organic matter (POM) and mineral‐associated organic matter (MOM) fractions in forest mineral soils (0–20 cm). Our results show consistent enrichment in ^13^C and ^15^N across all SOM fractions with increasing elevation, indicating a ubiquitous transformation state among SOM fractions regardless of environmental conditions. However, C stocks in the organic layer and in mineral soil POM increased proportionally relative to MOM with increasing elevation. Additionally, ^14^C‐based turnover times in the organic layer, the free POM, and the fine MOM fractions increased with elevation, indicating slower SOM processing and a reduced transformation of POM to MOM under harsher climatic conditions. In contrast to individual SOM fractions, total SOC stocks and ^14^C‐based turnover times in the bulk mineral soil showed no elevational pattern. This indicates that with increasing elevation, the shift in composition towards POM, which has a shorter turnover time than MOM, offsets the increased turnover time within each fraction. As a result, the overall SOC turnover time remains stable across the entire elevation gradient. However, the higher proportion of C stored in the more vulnerable POM fractions in high‐elevation forests indicates that their SOC stocks may be at higher risk under climate change.

## Graphical abstract

Climate change impact on soil organic carbon (SOC) pools remains uncertain. Our study sheds new light on the evolution of forest SOC stocks and their persistence (turnover times) along an elevation gradient serving as a proxy for climate variation. Despite the widespread use of soil organic matter fractionation, ^14^C studies of these fractions remain sparse. Our study of over 50 forest sites along 1700 m of elevation shows that longer turnover times are offset by higher proportions of fast‐cycling, unprotected particulate organic matter (POM) at higher elevations. This accumulation of POM at high‐elevation sites may be vulnerable to climate change.

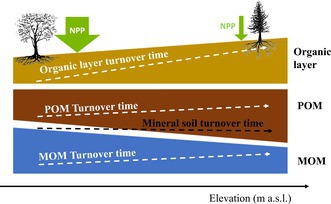

## 1 Introduction

Soils are the largest reservoir of organic carbon in terrestrial ecosystems, containing between 1700 and 2400 Gt C globally (Ciais et al. 2013; Friedlingstein et al. 2022). Soils are also considered to play an essential role in carbon sequestration (Basile‐Doelsch et al. 2020; Cotrufo et al. 2019), contributing to the uptake of 3.1 ± 0.6 Gt C year^−1^ by terrestrial ecosystems (Friedlingstein et al. 2022). However, despite decades of research on soil organic matter (SOM), the magnitude and factors regulating the storage and turnover of organic carbon (OC) in soils remain uncertain (Carvalho et al. 2024; Fromm et al. 2024; Lawrence et al. 2020).

Soil carbon storage is highly sensitive to climate change. For instance, increasing temperatures can accelerate microbial activity in the soil, increasing carbon losses through mineralization processes (Davidson and Janssens 2006; Guttières et al. 2021; Nissan et al. 2023). Conversely, a warmer climate may enhance plant productivity, leading to higher C inputs into soils (Djukic et al. 2010; Hagedorn et al. 2019). Deriving quantitative constraints on the net balance between carbon inputs and its mineralization remains challenging (Doetterl et al. 2025; Soong et al. 2021). Short‐term warming experiments in the laboratory and in the field have revealed a direct stimulation of soil organic carbon (SOC) turnover by increasing temperatures. However, C losses from soils may be ephemeral and decline within a few years due to the depletion of microbially accessible SOC as well as thermal acclimation of microbial communities (Eliasson et al. 2005; Melillo et al. 2017).

As an alternative approach to short‐term experimental manipulation, SOC cycling has been investigated along latitudinal gradients that encompass pronounced natural variations in climatic conditions (Doetterl et al. 2015; García‐Palacios et al. 2024; Wasner et al. 2024). Results show that climate effects on SOC cycling and storage depend on the interactions with other ecosystem properties such as soil physico‐chemical characteristics and vegetation (Doetterl et al. 2015; García‐Palacios et al. 2024; Wasner et al. 2024; Ziegler et al. 2017). While these gradients provide insights into long‐term adaptation processes, this approach introduces uncertainty due to the spatial heterogeneity of key driving factors (Haaf et al. 2021). Although less commonly used, elevation gradients in mountainous regions provide a similar natural set‐up to latitudinal gradients for exploring climate controls on SOC cycling (Djukic et al. 2010; Hagedorn et al. 2019; Khedim et al. 2023; Leifeld et al. 2009). While there is a clear increase in temperature with decreasing elevation, elevation‐related patterns in precipitation are less consistent. Nevertheless, forests in Central Europe are experiencing drought conditions more frequently at lower elevations (Meusburger et al. 2022). This is due to a combination of lower precipitation and higher temperatures, which together result in increased water loss through evapotranspiration.

At a global scale, estimates of soil C fluxes such as soil respiration and SOM mineralization show several‐fold decreases in C fluxes towards colder biomes (Nissan et al. 2023; Zhao et al. 2024). In comparison, modeled patterns of SOC stocks reveal similar high SOC stocks at high latitudes (Jungkunst et al. 2022). For certain regions and countries, SOC stocks may even exhibit opposing patterns, with decreases observed from temperate to boreal systems—for example, in Sweden (Fröberg et al. 2011) and Canadian boreal forests (Ziegler et al. 2017). Overall, the pronounced changes in carbon fluxes, contrasted with the more subtle variations in SOC stocks, indicate a balance between carbon inputs and outputs along climatic gradients. However, the controlling factors behind this balance remain poorly constrained at both landscape and global scales (Doetterl et al. 2015; Haaf et al. 2021; Soong et al. 2021).

One reason for the difficulty in assessing climate change impacts on SOM is its heterogeneous nature, encompassing simple to complex compounds that turn over on daily to millennial time scales, and on diverse spatial scales (González‐Domínguez et al. 2019; van der Voort et al. 2017). To capture its dynamics, SOM can be separated into operationally defined fractions, aiming at representing pools with different properties and turnover times (Heckman et al. 2022). One common approach involves separation according to SOM density, with the lower‐density (“light”) fraction representing predominantly mineral‐free particulate organic matter (POM), and the higher‐density fraction comprising mineral‐associated organic matter (MOM) (Gies et al. 2021; Griepentrog et al. 2014; Lavallee et al. 2020). These fractions reflect the current view of SOM transformation where plant‐derived OM enters the soil as POM, which is then subject to continuous processing by microbial communities (Lehmann and Kleber 2015). The resulting microbial residues are stabilized through interactions with reactive mineral surfaces. This process, known as microbial entombment (Cotrufo et al. 2015; Liang et al. 2017), along with the sorptive stabilization of dissolved organic matter on mineral surfaces (Kalbitz et al. 2005), can preserve soil organic matter (SOM) for centuries or even millennia (Gies et al. 2021; Heckman et al. 2022). Density fractionation of SOM shows increasing contributions of POM relative to MOM towards colder climates (García‐Palacios et al. 2024). This trend has been attributed to slower transformation of plant‐derived carbon inputs and reduced transfer to MOM at lower temperatures, leading to higher proportions of POM, which is more vulnerable to disturbances. Unlike intensively studied agricultural soils, POM in forest soils accumulates either as an organic layer on top of the mineral soil or within the mineral soil itself. Only a few larger‐scale soil fractionation studies have included the organic layer in their assessment and, if so, the transformed F and H horizons of the organic layer were combined with the mineral soil (Cotrufo et al. 2015), omitting an important part of the C cycling in forest soils.

Carbon and nitrogen isotopic ratios (Δ^14^C, δ^13^C and δ^15^N) provide insights into SOM transformation and turnover. Due to the preferential use of light isotopes by microbial communities, there is an enrichment in ^13^C and ^15^N during SOM transformation (Conen et al. 2008; Kramer et al. 2003; Lorenz et al. 2020). Radiocarbon (^14^C) has proven to be a uniquely powerful approach to estimate turnover times of different soil pools, allowing determination of C cycling rates and C exchange between different reservoirs (Brunmayr et al. 2024; Grant et al. 2024; Schuur et al. 2016; Trumbore 2000). The ~5700‐year half‐life of ^14^C allows assessment of processes occurring on centennial to millennial time scales (Trumbore 2000) while the abrupt ^14^C signal (“bomb spike”) induced by nuclear weapons testing in the mid‐20th century can be used to track the incorporation and turnover of C within different reservoirs on (sub‐)decadal time scales (Eglinton et al. 2023). Global syntheses of ^14^C data of bulk soils have revealed slower C turnover towards colder biomes. The mean soil C ages in these biomes were older than predicted by current Earth system models, highlighting limitations in our understanding of mechanisms that control C persistence in soils (Brunmayr et al. 2024; He et al. 2016; Shi et al. 2020). Although SOM fractionation has been widely applied (Conen et al. 2008; Griepentrog et al. 2014; Schrumpf et al. 2021), ^14^C studies of SOM fractions in forest soils remain limited and are generally based on only a few sites (Fröberg et al. 2011; Porras et al. 2017).

Here, we examine SOM cycling and C storage across a gradient spanning sub‐Mediterranean to alpine forests in Switzerland, using elevation as an integrating variable for climate variation, vegetation composition, and soil properties. Our goal was to assess variations in stocks, transformation state, and turnover times of SOM fractions of forest soils, encompassing POM in the organic layer and mineral soil, as well as various MOM fractions. To determine SOM composition and transformation, we fractionated SOM using density and size separation, as well as chemical oxidation, and analyzed corresponding stable isotopic signatures. We also assessed turnover times of SOM and its fractions based on ^14^C contents. We anticipated that the transformation of C inputs would slow with increasing elevation, and thus with harsher climatic conditions under the increasing prevalence of coniferous forests. We hypothesized that this deceleration would shift the dominant pathways of SOM cycling, with a greater accumulation of POM in high‐elevation forest soils due to a slower processing of SOM inputs. Simultaneously, we expected slower and reduced formation of stabilized MOM due to a decreased microbial entombment. We examined how these opposing effects manifest themselves in relation to overall carbon stocks and turnover times.

## 2 Methods

### 2.1 Site Selection and Site Characteristics

**FIGURE 1:**
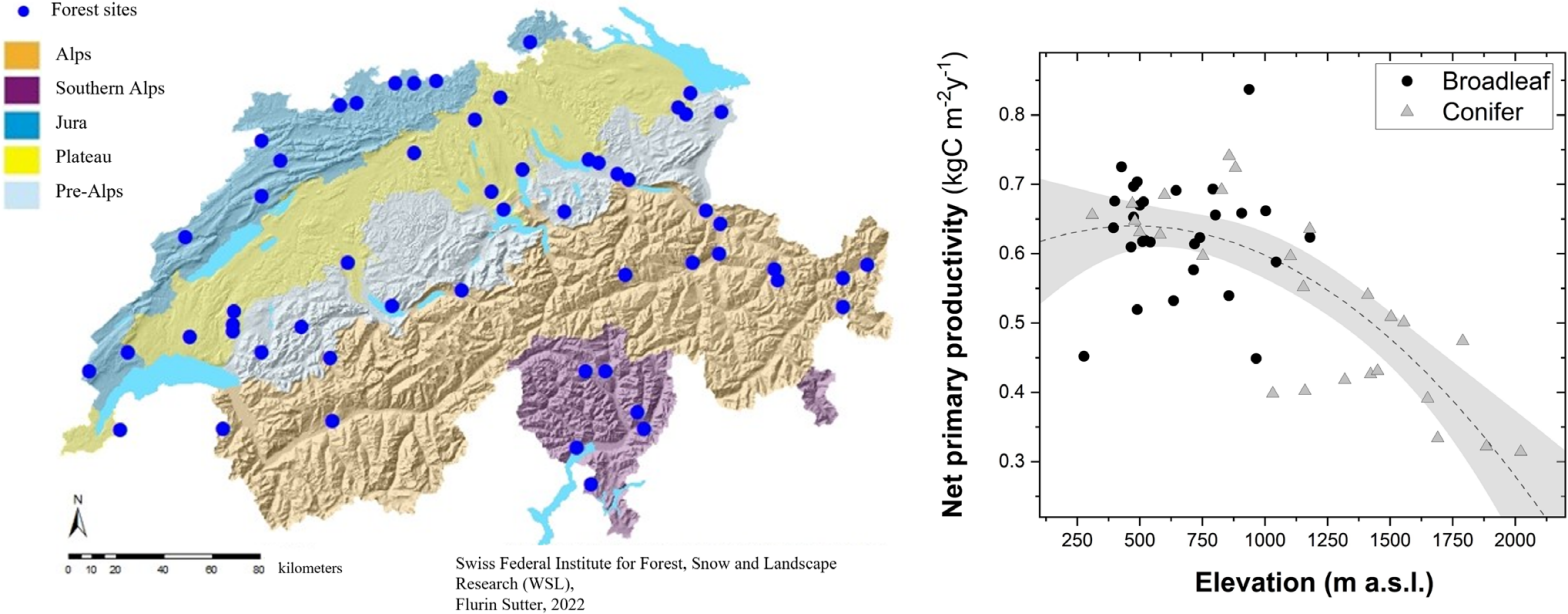
The 54 forest sites across the five regions of Switzerland (left), covering an elevation gradient with a declining net primary productivity (*R* ^2^ = 0.54; *p* < 0.001) and an increasing contribution of coniferous trees with increasing elevation. Map lines delineate study areas and do not necessarily depict accepted national boundaries.

For this study, 52 sites with a continuous forest cover for over 120 years were chosen from the Swiss Federal Institute for Forest, Snow and Landscape Research (WSL) soil database that comprises approximately 1000 sites (Gosheva 2017). The selection was done following a stepwise selection approach that considered climatic variables, soil properties, and site features (González‐Domínguez et al. 2019). The selected sites cover an elevation gradient from 270 to 2020 m a.s.l. and are evenly distributed across four climatic classes defined by site temperatures and by a dryness index (González‐Domínguez et al. 2019), encompassing the five regions of Switzerland, that is, the Jura, Plateau, Pre‐Alps, Alps, and Southern Alps (Fischer and Traub 2019) (Figure 1). We sampled two additional high‐elevation sites at the treeline (Hagedorn et al. 2010) and in a subalpine forest (Hiltbrunner et al. 2013).

For each site, we averaged the mean annual temperature (MAT) and mean annual precipitation (MAP) over the period 1981–2010. Potential evapotranspiration (PET) was also averaged over the same period according to Penman‐Monteith (Remund et al. 2014). To obtain a proxy for litter inputs at each site, we retrieved the average net primary production (NPP) (Figure 1) for the period 2001–2022 from Terra and Aqua MODIS satellite at 500‐m resolution (Running and Zhao 2021a, 2021b). The leaf area index (LAI) of trees and shrubs was measured using hemispherical photography (Schleppi et al. 2011). Soil characteristics, such as texture, pH, exchangeable cations, and pedogenic oxides were obtained from the WSL soil database (Walthert et al. 2013) and measured in the bulk mineral soil following methods from Walthert et al. (2004).

### 2.2 Soil Sampling

In 2014, a soil corer of 5 cm in diameter was used to sample the organic layer and the underlying mineral soils from 0 to 20 cm depth at each of the 52 sites (González‐Domínguez et al. 2019). To account for the site‐specific spatial variability, eight cores were taken from three nonoverlapping 40 m x 40 m subplots at each site, with a total of 24 cores per site. The organic layer was sampled as a whole and then separated into L, F, and H layers according to its characteristics (FAO 2014) in the lab. From the 24 cores from each site, one composite sample for the organic layers and one for the mineral soil were obtained. The samples were stored at 4°C upon return from the field. The mineral soil was then oven‐dried at 40°C and sieved by hand (≤ 2 mm) (González‐Domínguez et al. 2019). At the two additional sites, three soil profiles were excavated, and organic layer as well as 0–20 cm depth mineral soil samples were collected.

### 2.3 Density and Chemical Fractionation

The mineral soil samples were separated into four soil density fractions following the methodology of Cerli et al. (2012) and Griepentrog et al. (2014): free light fraction (fLF), occluded light fraction (oLF), coarse heavy fraction (cHF), and fine heavy fraction (fHF) (Müller et al. 2015). To separate the fLF, 40 mL of a solution of sodium polytungstate (SPT) with a density of 1.6 g/cm^3^ was added to 10 g of soil in a 50 mL Falcon tube. The density of 1.6 g/cm^3^ was chosen to separate a pure organic (mineral‐free) light fraction, following pretests (Cerli et al. 2012). After centrifugation (10 min at 4500 g), the fLF was carefully removed and placed on glass‐fiber filters (GF 6, Whatman, d = 47 mm; nominal pore size 300 μm) and rinsed with Milli‐Q water. The remaining soil was then ultrasonically dispersed in 40 mL of SPT, applying an energy of 300 J/mL (UW 3400, Bandelin, Berlin, Germany). After centrifugation, the floating oLF was collected, transferred to a filter, and rinsed. The material remaining was separated into fHF and cHF by wet sieving at 20 μm. We here consider that the fLF and the oLF represent the POM, whereas the cHF and fHF represent the MOM.

To gain additional insights into the turnover and stability of MOM, and to access the older and well stabilized C, the fine heavy fraction was chemically oxidized with hydrogen peroxide (H_2_O_2_) over 1 week, according to Favilli et al. (2009) and Schrumpf et al. (2021), whereby peroxide oxidation is considered to simulate oxidative processes occurring in the soil. In brief, a total of 90 mL H_2_O_2_ (10%) was added to 1 g of dry fHF in increments of 30 mL after an initial rewetting of the sample with 10 mL of Milli‐Q water. The samples were placed on a stirring plate at 50°C to catalyze the reaction. After the oxidation reaction was completed, the samples were freeze‐dried. Combusted sand was used as a processing blank to track the potential contamination through the oxidation process.

### 2.4 C, N Concentrations and Stocks

The total concentrations and stable isotope ratios of C and total N were measured by an EA/IRMS (Euro‐ EA 3000, HEKAtech GmbH, Germany, interfaced with a Delta‐V Advanced IRMS, Thermo GmbH, Germany). The measurement uncertainty on the isotope ratios was lower than 0.30‰. The samples were ground for 3 min using a ball mill (Retsch MM2000) before measurement. Samples with pH > 6 were fumigated with HCl following the method described in Walthert et al. (2010) to remove carbonates. SOC stocks were calculated by multiplying the fine earth density by the OC content (%) taking into account field estimates of stone contents (Gosheva et al. 2017). Additionally, the organic layer and the total SOC stocks down to the bedrock, at our 50 sites, were obtained from the WSL database (Walthert et al. 2013).

### 2.5 Stable Isotope Enrichment Factor

We calculated the ^15^N enrichment factor (*ε*[‰]) from the microbial processing of organic matter between the POM and the fHF as follows (Vervaet et al. 2002):

$$\varepsilon \left[‰\right]=\frac{\delta^{15}N_{\mathrm{POM}}-\delta^{15}N_{\mathrm{fHF}}}{\ln\left(N_{\mathrm{POM}}\right)-\ln\left(N_{\mathrm{fHF}}\right)}$$

with $\delta^{15}N_{\mathrm{POM}}$ and $\delta^{15}N_{\mathrm{fHF}}$ being the isotopic ratios for the POM and the fHF, respectively and *N*_*POM*_ and *N*_*fHF*_ are the nitrogen contents in the POM and fHF expressed in per cent. $\delta^{15}N_{\mathrm{POM}}$ and $N_{\mathrm{POM}}$ were obtained through the following mixing models:

$$\delta^{15}N_{\mathrm{POM}}=\delta^{15}N_{\mathrm{fLF}}*w_{\mathrm{fLF}}+\delta^{15}N_{\mathrm{oLF}}*w_{\mathrm{oLF}}$$

$$N_{\mathrm{POM}}=N_{\mathrm{fLF}}*w_{\mathrm{fLF}}+N_{\mathrm{oLF}}*w_{\mathrm{oLF}}$$

with $w_{\mathrm{fLF}}$ and $w_{\mathrm{oLF}}$ being the contribution of fLF and oLF, respectively to the total N stock in POM.

The ^13^C enrichment factor was calculated in a similar way:

$$\varepsilon \left[‰\right]=\frac{\delta^{13}C_{\mathrm{POM}}-\delta^{13}C_{\mathrm{fHF}}}{\ln\left(C_{\mathrm{POM}}\right)-\ln\left(C_{\mathrm{fHF}}\right)}$$

with *δ* ^*13*^ *C*_*POM*_ and *δ* ^*13*^ *C*_*fHF*_ being the carbon stable isotope ratios of the POM and the fHF and the *C*_*POM*_ and *C*_*fHF*_ being the OC contents (in %) in the POM and fHF, respectively.

### 2.6 Radiocarbon Measurements

^14^C was measured in the organic layers, bulk mineral soil, density fractions, and in the residual fraction after H_2_O_2_ oxidation using an elemental analyzer coupled to a MICADAS (Miniaturized radioCArbon DAting System, Ionplus AG) equipped with a gas‐accepting ion source at the Laboratory of Ion Beam Physics, ETH Zürich (McIntyre et al. 2017; Ruff et al. 2010; Synal et al. 2007). Prior to the radiocarbon measurement, the samples were fumigated with HCl (37%) in a desiccator at 60°C (72 h) to remove inorganic carbon and then neutralized by exposure to NaOH (Komada et al. 2008). Two reference samples (shale and an internal reference soil from Othmarsingen) were always fumigated along with sample batches to detect and correct for potential contamination during the fumigation process. To correct for potential contamination during the H_2_O_2_ oxidation process, we included combusted sand as a process blank. We assumed a constant contamination and applied the constant contamination correction (Haghipour et al. 2019). The contamination mass was on average 7 μg of C and had a F^14^C of 0.5. The radiocarbon content of the H_2_O_2_‐oxidized fraction (^14^C_ox_) was obtained by computing a mass balance.

$$\mathrm{mO}C_{\mathrm{ox}}=\mathrm{mO}C_{\mathrm{fHF}}-\mathrm{mO}C_{\mathrm{res}}$$

Where *mOC*_*ox*_ is the mass of organic carbon that was removed from the sample by the hydrogen peroxide. *mOC*_*fHF*_ is the initial mass of organic carbon, in the fine heavy fraction. *mOC*_*res*_ is the mass of carbon present in the residual fraction after H_2_O_2_ oxidation.

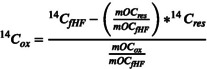




Where ^*14*^ *C*_*fHF*_ is the radiocarbon content in the fHF and ^*14*^ *C*_*res*_ the is radiocarbon content in the residual fraction after H_2_O_2_ oxidation.

In our radiocarbon dataset, we included the Δ^14^C values from respired CO_2_ during soil incubation in the laboratory as well as the bulk soil Δ^14^C values measured by González‐Domínguez et al. (2019).

### 2.7 Turnover Time Modeling

To compare Δ^14^C results between different fractions and across sites while integrating the effect of the bomb spike on Δ^14^C, we used a one pool model under the assumptions that each fraction is a homogenous carbon pool under steady state. For each SOC fraction, we computed the ^14^C‐derived turnover time, defined as the ratio between the C stocks and the input or output flux (Sierra et al. 2017). The ^14^C‐derived turnover time *τ* is the inverse of the SOC decomposition rate *k* (i.e., *τ = 1/k*) (González‐Domínguez et al. 2019). We modeled the ^14^C‐derived turnover time for each fraction separately because the exact carbon input pathway into each fraction is unknown and difficult to assess. The parallel modeling enables us to compare the turnover times between sites and fractions among them since they are independently modeled. We applied the following model simulation (Torn et al. 2009):

$$\frac{d\left(F*C\right)}{\mathrm{dt}}=I*F_{\mathrm{atm}}-\left(k+\lambda \right)\left(F*C\right)$$

Where *I* is the input of C into the C pool, *F*_*atm*_ is the fraction modern of the atmosphere, *k* is the SOC decomposition rate, *λ* (= 1.21 × 10^−4^ year^−1^) is the radioactive decay rate of ^14^C equal to ln(2)/5730. *F* is the absolute fraction modern of the pool, defined as (González‐Domínguez et al. 2019; Schuur et al. 2016):

$$F=1+\left(\frac{\Delta^{14}C}{1000}\right)$$

We used the function OnepModel14() from the *SoilR* package (version 1.2.107) (Sierra et al. 2014) to simulate F^14^C values over the period 1850 to 2019 using atmospheric F^14^C data from IntCal20 Reimer et al. (2020) and from Hua et al. (2022) from the NH1 zone, and for the years 2020 until 2022 we averaged the ^14^C data from the Jungfraujoch (Emmenegger et al. 2023). We introduced a time lag of respectively 2, 5, and 8 years for the deciduous, mixed, and coniferous forests based on previous studies that found a turnover time of 2 years in their deciduous litterfall and 8 years in the F layer (Harkness et al. 1986; Van Der Voort et al. 2019). We iteratively tested decomposition rates corresponding to ^14^C‐derived turnover times from 1 to 10,000 years. We then searched for the *k* value that had the minimum absolute difference between simulated and measured F^14^C values. For the samples where Δ^14^C > 0, we looked for two minima since the ^14^C‐derived from the bomb spike which can be either from the ascending or descending limb of the curve. To disentangle these two possible turnover times, we calculated the input flux into the pool for the two possible solutions (Torn et al. 2009):

$$\text{flux}=\frac{C_{\text{stock}}}{\tau }$$

We then selected the turnover time that best conformed to additional criteria. Specifically, for the organic layers and the fLF, we verified that the flux was smaller than half of the NPP. Fröberg et al. (2011) included in their model an OC flux into the humus layer that accounted for 47% of the total annual litter input. If both turnover time solutions fulfilled this condition, we chose the shorter turnover time because it was the most realistic given that the fLF is mainly plant derived. Eleven sites had a turnover time above 80 years in the organic layer and fLF, which was deemed unrealistic and an artifact of the model; these sites were removed from the analysis. One of these sites had a high content of fire derived pyrogenic C (González‐Domínguez et al. 2019) which is known to complicate turnover time modeling (Leifeld 2008). For the oLF, if both turnover time solutions satisfied this condition, we chose the longer turnover time because the oLF reflects C stabilized in aggregates. Given that we applied a relatively strong sonication energy (300 J ml^−1^) to disrupt the aggregates, this treatment should release C that has been subject to physical protection (Griepentrog et al. 2014; Schmidt et al. 2011). For the MOM fractions, we always chose the longer turnover times, assuming that in the MOM fractions the C is preserved over long periods of time. Similarly, for the bulk mineral soil, we checked that the flux was not higher than the NPP and selected the longer turnover times, which were more realistic.

### 2.8 Data Evaluation and Statistical Analysis

All statistical analyses were carried out in R 4.2.2 (R Core Team 2022). To characterize ecosystem properties along the studied elevation gradient, we computed the Pearson correlation coefficient between elevation and a set of variables, including climate, vegetation, and soil properties. We investigated fixed effects of elevation and mineral SOM fractions on SOC stocks, the C/N ratio, δ^13^C, δ^15^N and Δ^14^C values, and ^14^C‐based turnover times, including the site as a random effect, through linear mixed‐effects models in the lmerTest package in R (Kuznetsova et al. 2017). The variables were scaled prior to running the models. Elevation was log‐transformed to comply with the assumed normal distribution of model residuals. Furthermore, we ran linear models to investigate the effect of elevation on the response variables in the different fractions. All models were considered statistically significant when the *p*‐values of the model *F*‐statistics were below 0.05. We visually checked the residual plot from the models to validate model assumptions. In our data analysis, we identified four sites out of the 54 sites which had a POM/MOM ratio above the third quartile plus three interquartile ranges, using the is_extreme () function from the *rstatix* package (Kassambara 2023), and removed them from our analysis. Two of these sites were waterlogged, while the other two sites were dry sites with a Xeromoder type organic layer overlaying the mineral soil with high stone content. Our final dataset contained 50 sites. For the F and H layers and the fLF fraction, the highest site at treeline had very long turnover times (151, 168 and 1182 years, respectively). As the leverage plot showed that this site was driving the elevation relation, we removed it from the linear model of turnover time against elevation. One site had petrogenic‐derived C in the cHF and residual fraction. The leverage plot from the linear models of elevation effect on turnover time in the cHF and in the residual fraction showed this site as an outlier, so we removed this site from these models.

## 3 Results

### 3.1 Elevation Gradients

Across Swiss forest ecosystems, site elevation is negatively correlated with mean annual temperature (MAT) but not with mean annual precipitation (Table 1), which peaks around 1300 m a.s.l. and declines towards lower and higher elevations. Net primary productivity (NPP) obtained from MODIS decreases by approximately 50% from 0.75 to 0.31 kg C m^−2^ year^−1^. Also, the leaf area index (LAI) as well as the fraction of broadleaf trees declines with elevation along the gradient encompassed by our sites (Figure 1). Soil pH and contents of clay, calcium, and Al oxides did not exhibit any significant pattern with elevation, while Fe‐oxides show a tendency to increase towards high elevation (Table 1).

**TABLE 1:** Climatic conditions, vegetation and soil properties along the elevation gradient across the 50 Swiss forest sites. Pearson correlation coefficients between the different variables and elevation, with statistically significant relationships highlighted in bold.

	Mean annual temperature	Mean annual precipitation	Potential evapotranspi‐ration	Leaf area index	Percentage of deciduous trees	Net primary productivity	pH	Clay	Calcium content	Al_oxa_ oxides	Fe_oxa_ oxides	Mn_oxa_ oxides
	°C	mm	mm	—	%	kg C m^2^ year^−1^		%	mmolc kg^−1^	g kg^−1^	g kg^−1^	g kg^−1^
Minimum	1.1	704	356.2	2.5	0	0.31	3.0	4.8	1.2	0.0	0.013	0.000
Median	8.0	1300	468.6	4.3	52	0.62	4.6	20.2	99.6	1.5	3.091	0.300
Maximum	11.8	2216	601.2	6.3	100	0.74	7.6	60.2	942.3	28	32.294	2.431
Pearson correlation coefficient with elevation	**−0.97**	0.09	**−0.70**	**−0.74**	**−0.62**	**−0.72**	−0.15	−0.09	0.01	0.19	**0.26**	0.02
*p*	**< 0.001**	n.s	**< 0.001**	**< 0.001**	**< 0.001**	**< 0.001**	n.s	n.s	n.s	n.s	**0.07**	n.s

### 3.2 Soil Organic Carbon Stocks

**FIGURE 2:**
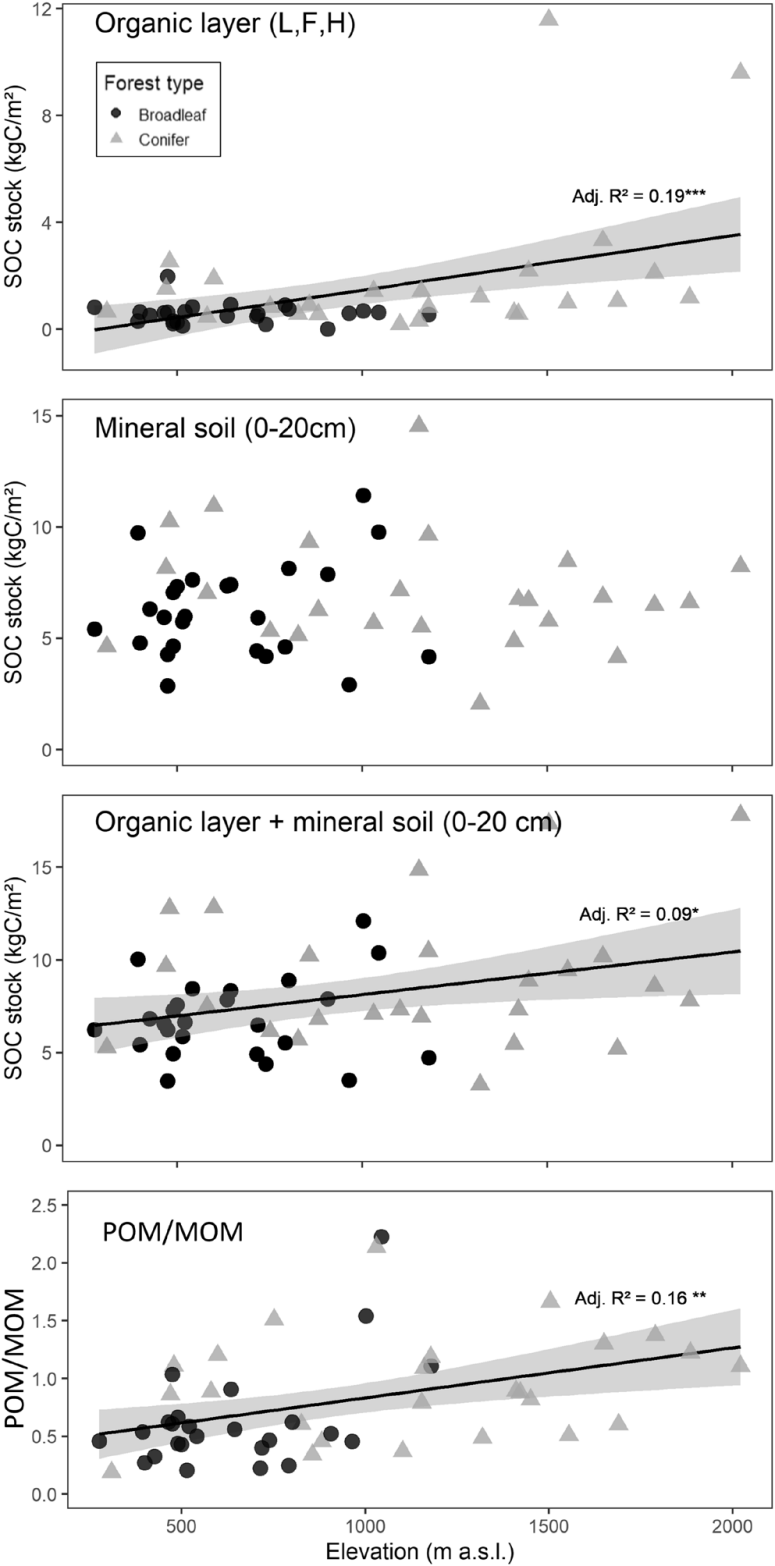
Organic carbon stocks (kg C/m^2^) are presented along the elevation gradient for the total organic layer (L, F, H), the top 20 cm of mineral soil, and the combined profile (organic layers + top 20 cm of mineral soil). The bottom graph presents the elevation pattern of the ratio between the particulate organic carbon stock and the mineral‐associated organic carbon stock (POM/MOM) in the top 20 cm of mineral soil.

**FIGURE 3:**
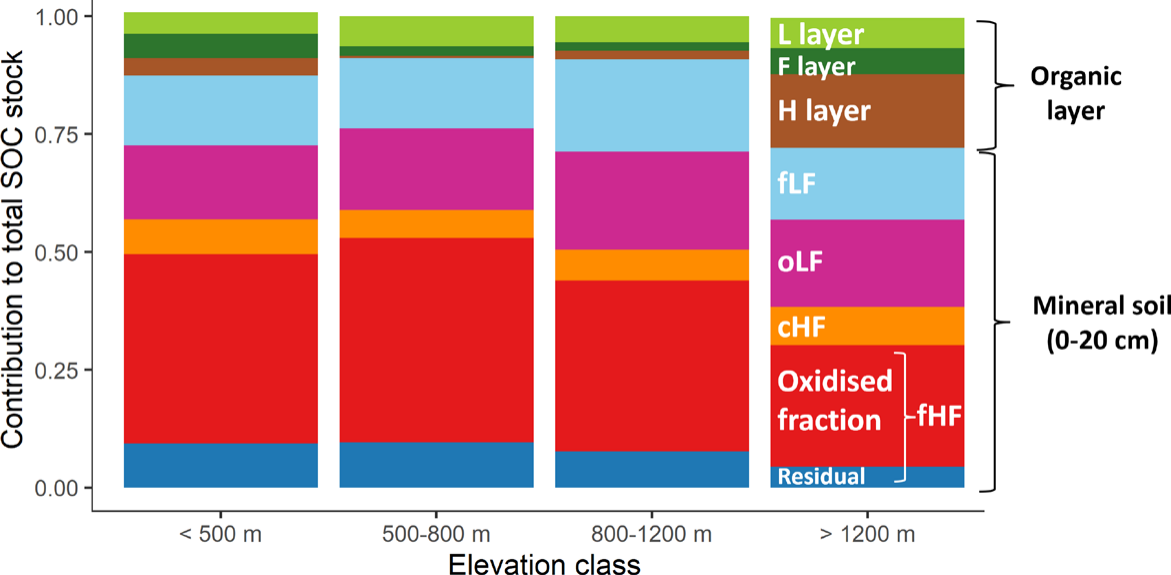
Contribution of the different soil fractions to the total SOC stock (organic layer and mineral soil, 0–20 cm). The free light fraction (fLF) and the occluded light fraction (oLF) are the two particulate organic matter fractions. The mineral‐associated organic matter fractions are the coarse heavy fraction (cHF), hydrogen peroxide‐oxidized fraction (oxidized fraction) and the remaining fraction after the hydrogen peroxide oxidation (residual fraction).

In the organic layer, SOC stocks of the 50 sites showed a significant increase with elevation (*p* < 0.001), reaching up to 11.6 kg C m^−2^ (Figure 2). In contrast, SOC stocks in the mineral soil at 0–20 cm depth, ranging from 2 to 22 kg C m^−2^ across our 50 sites, were not correlated to elevation (*p* > 0.05). There was a weak positive correlation between elevation and total C stocks (organic layer plus mineral soil 0–20 cm) (*p* = 0.02) increasing by 2.3 kg C m^−2^ per 1000 m rise in elevation. This reflects the increasing contribution of the organic layer to total SOC stocks with elevation (*p* < 0.001) with an increase of 2.0 kg C m^−2^ per 1000 m rise in elevation. At elevations above 1200 m a.s.l., 25% of the SOC was stored in the organic layer, whereas at low‐elevation sites (< 500 m a.s.l), the organic layer represented only 12% of the total SOC stock (Figure 3). Total SOC stocks of the 50 sites down to the bedrock averaged 15.3 ± 2.7 kg C m^−2^ and did not show a significant linear relation with elevation (*p* = 0.17). For a larger data set of 556 Swiss sites with a reconstructed forest cover for more than 120 years (Gosheva 2017), total SOC stocks remained unrelated to elevation while the contribution of the organic layer to total SOC stocks was higher in coniferous than in broadleaf forests and increased significantly with elevation (*p* < 0.001; Figure S1). In the mineral soil at 0–20 cm depth, POM (fLF+ oLF) comprised on average 41% of the total SOC stocks (Figure 3). Among MOM fractions, the cHF represented only 8% of total SOC, while the oxidized and residual fraction of the fine MOM contained 42% and 9% of the total SOC stock in the 0–20 cm mineral soil, respectively (Figure 3). The relative contribution of POM increased with elevation (*p* < 0.05) from 35% at elevations lower than 500 m a.s.l. to 47% above 1200 m of elevation (Figure 3), while the proportion of MOM decreased along the same trajectory (Figures 2 and 3, Table 2 and 3).

**TABLE 2:** Analysis of variance of the effects of elevation and fractions on SOC stocks at 0–20 cm depth, C/N ratio, δ^13^C, δ^15^N, Δ^14^C, ^14^C‐derived turnover times tested with linear mixed‐effects models with site location as a random effect.

Fixed effects	SOC stocks	C/N	δ^13^C	δ^15^N	Δ^14^C	Turnover times
Elevation	n.s.	**	***	*	n.s.	*
Fractions	***	***	***	***	***	***
Elevation × Fractions	***	*	n.s.	n.s.	n.s.	n.s.
*R* ^2^	0.62	0.62	0.46	0.87	0.64	0.78

*Note:* *p*‐values < 0.05 are marked with *, *p*‐values < 0.01 with **, *p*‐values < 0.001 with ***.

**TABLE 3:** Linear models testing the effect of elevation on the POM/MOM ratio and the SOC stocks in the different fractions. The table reports the slope directions of the linear regressions against elevation, the *R* ^2^ and the *p* values from the models, significant relationships are highlighted in bold. The plus signs in red show an increase of the SOC stocks along elevation whereas the minus signs in blue show a decrease of the SOC stock with elevation.

		SOC stocks (kg C/m^2^)									
	POM/MOM	L layer	F layer	H layer	Bulk mineral soil (0–20 cm)	fLF	oLF	cHF	fHF	Oxidized fraction	Residual fraction
Elevation	+	n.s	n.s	+	n.s	n.s	+	n.s	**−**	**−**	**−**
Adj. *R* ^2^	**0.17**	0.002	< 0	**0.15**	< 0	0.02	**0.08**	0.002	**0.09**	**0.06**	**0.14**
*p*	**0.002**	n.s	n.s	**0.004**	n.s	n.s	**0.02**	n.s	**0.02**	**0.049**	**0.005**

### 3.3 Soil Organic Matter Composition

**FIGURE 4:**
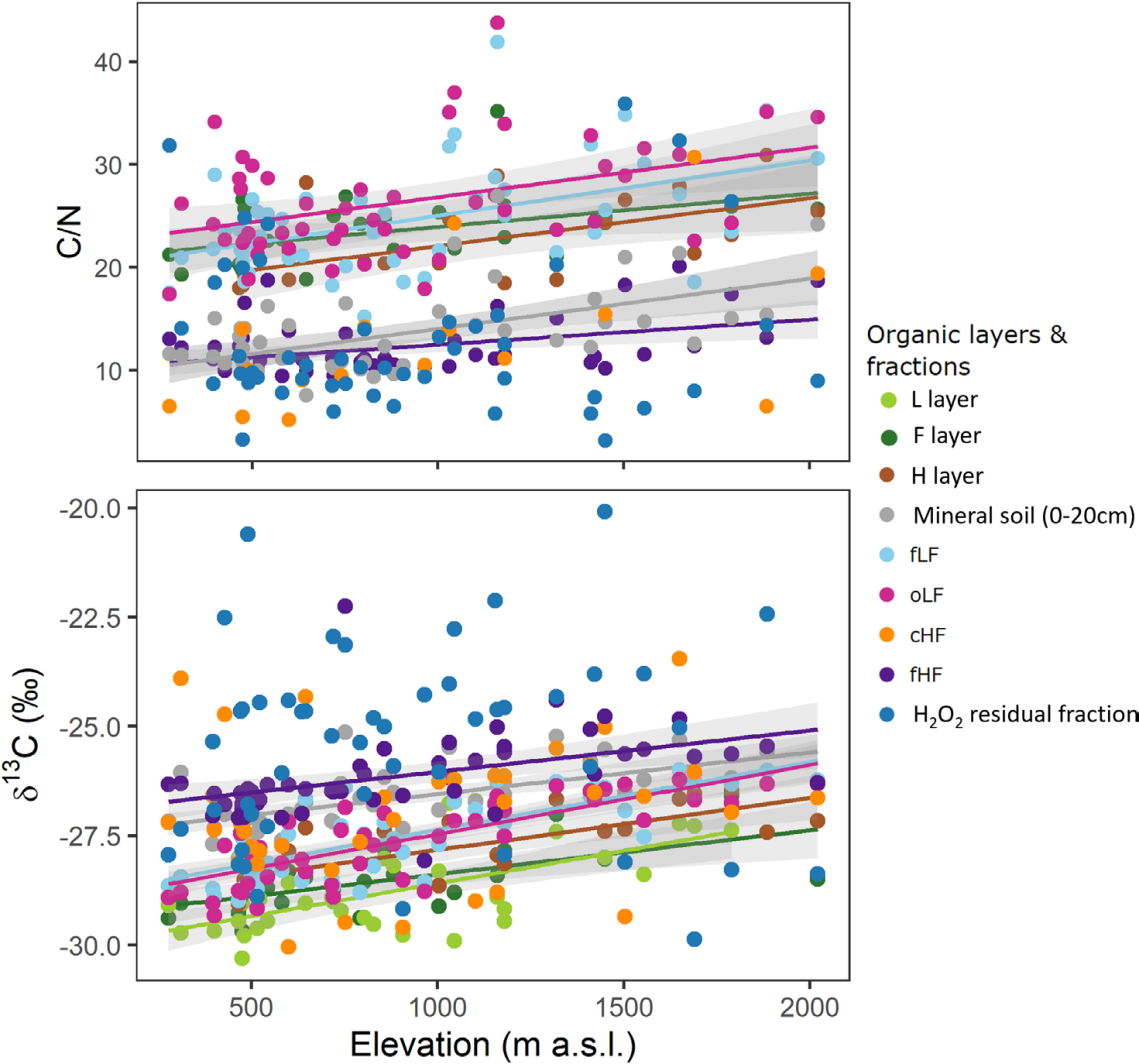
Linear relations between elevation and C/N (top graph) and δ^13^C values (‰) (bottom graph) in the soil organic carbon fractions. Only significant relations are shown.

Both δ^13^C and δ^15^N values showed significant increases from the organic layers and roots to POM (fLF and oLF) and MOM (cHF and fHF) (Figure 4 and Figure S4). Also, the C/N ratio decreased from plant residues to MOM. Across all sites and all fractions, there was an average ^13^C enrichment factor ε^13^C of 0.49‰ and an ε^15^N of 2.30‰ from the organic layers to MOM (corresponding to the slopes in Figure S4). Both enrichment factors were constant along the elevation gradient (Figure S5). Across the 50 Swiss forest soils, δ^13^C values and C/N ratios increased significantly with elevation in all fractions except in the H_2_O_2_ residual fraction and cHF for the C/N ratio (*p* < 0.05, Figure 4, Table 4). The δ^15^N values increased with elevation in the fLF, oLF, and fHF (*p*‐values < 0.01, Table 4 and Figure S3). The slopes of the linear regressions between elevation and δ^13^C and C/N ratio were similar for the different fractions (Figure 4). The δ^15^N slopes as a function of elevation were also similar for the POM fractions, the bulk mineral soil, and fHF (Figure S2).

**TABLE 4:** Linear models testing the effect of elevation on the C/N, δ^13^C, δ^15^N, Δ^14^C, and ^14^C‐based turnover, in the different fractions. The plus signs in red show an increase with elevation, whereas the blue minus signs indicate a decrease. Statistically significant relationships are highlighted in bold.

	L layer	F layer	H layer	Bulk mineral soil (0–20 cm)	fLF	oLF	cHF	fHF	H_2_O_2_ oxidized fraction	H_2_O_2_ residual fraction
C/N										
Slope	+	+	+	+	+	+	+	+		n.s
*R* ^2^	**0.07**	**0.17**	**0.29**	**0.24**	**0.20**	**0.13**	**0.26**	**0.086**	N.A	< 0
*p*	**0.04**	**0.02**	**< 0.01**	**< 0.01**	**< 0.01**	**< 0.01**	**0.02**	**0.02**		0.7
δ^13^C										
Slope	+	+	+	+	+	+	**n.s**	+		n.s
*R* ^2^	**0.37**	**0.38**	**0.60**	**0.40**	**0.56**	**0.62**	< 0	**0.22**	N.A	< 0
*p*	**< 0.01**	**< 0.01**	**< 0.01**	**< 0.01**	**< 0.01**	**< 0.01**	0.4	**< 0.01**		0.4
δ^15^N										
Slope	n.s	n.s	n.s	n.s	+	+	n.s	+		n.s
*R* ^2^	0.01	< 0	0.07	0.02	**0.07**	**0.08**	< 0	**0.07**	N.A	0.06
*p*	0.2	0.9	0.1	0.14	**0.04**	**0.03**	0.5	**0.03**		0.05
Δ^14^C										
Slope		n.s	n.s	n.s	n.s	n.s	n.s	**−**	**−**	n.s
*R* ^2^	N.A	< 0	0.1	< 0.01	< 0	< 0	< 0	**0.08**	**0.12**	< 0.001
*p*		0.5	0.09	0.2	0.999	0.4	0.5	**0.03**	**0.01**	0.3
Modeled turnover times										
Slope		n.s	n.s	n.s	+	n.s	n.s	+	+	+
*R* ^2^	N.A	0.05	0.17	0.04	**0.15**	< 0	< 0	**0.11**	**0.1**	**0.07**
*p*		0.1	0.1	0.08	**0.007**	0.8	0.7	**0.01**	**0.009**	**0.04**

### 3.4 Radiocarbon‐Based Turnover Times in SOM Fractions

Radiocarbon contents, expressed as Δ^14^C values, decreased from the organic layers and POM to MOM (Figure S6). Computing the ^14^C‐derived turnover time for SOC fractions yielded median C turnover times of 9 years in the F‐layer (mean ± standard deviation = 15.6 ± 5 years) and 17 years in the H‐layer (mean = 30 ± 11 years). The median turnover time for the bulk mineral soil was 131 years (mean = 171 ± 27 years). While the fLF had decadal turnover times (median = 12 years, mean = 67 ± 26 years), the oLF turned over on centennial time scales (median = 102 years, mean = 145 ± 27 years). In contrast, the cHF exhibited a broad range of turnover times from ~95 to ~8300 years (median = 469, mean = 856 ± 181 years). The H_2_O_2_ resistant fraction was depleted in ^14^C compared to the initial fHF and was turning over on a millennial timescale (median = 1009, mean = 1574 ± 279 years). The Δ^14^C value of the H_2_O_2_‐oxidized fraction, which was obtained via isotope mass balance, was enriched in ^14^C and yielded turnover times with a median of 134 years (mean = 170 ± 29 years).

**FIGURE 5:**
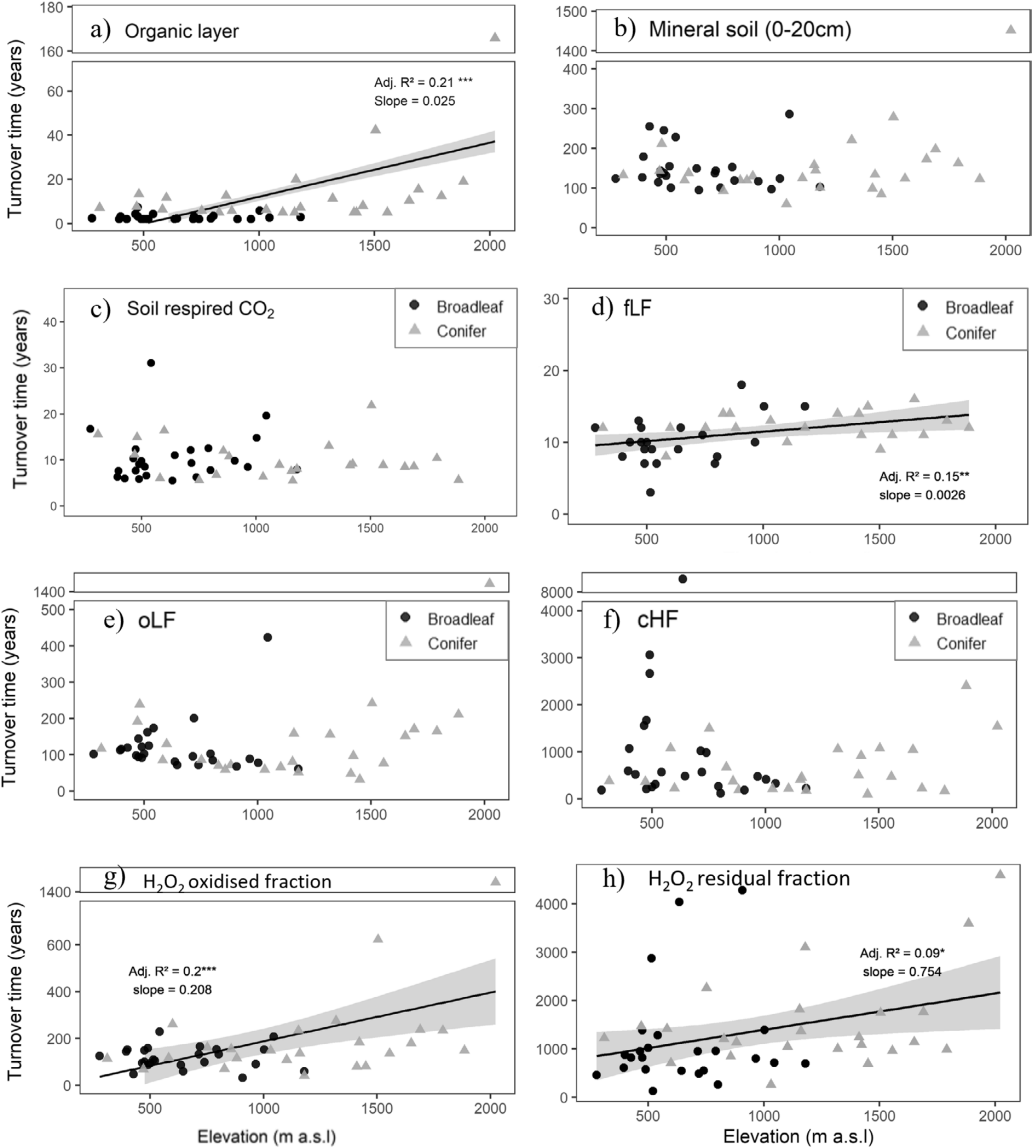
^14^C‐derived turnover times (in years) under steady state along elevation in (a) the organic layers, (b) bulk mineral soil from 0 to 20 cm depth, (c) respired CO_2_, (d) free light fraction (fLF), (e) occluded light fraction (oLF), (f) coarse heavy fraction (cHF), (g) in the H_2_O_2_ oxidized fraction and (h) the H_2_O_2_ residual fraction. The turnover time of the soil‐respired CO_2_ was obtained from González‐Domínguez et al. (2019). Slopes are expressed in years per m of elevation.

The ^14^C‐based turnover times increased significantly with increasing elevation in the organic layer, the fLF, and the oxidized and residual fHF fraction (Figure 5). In contrast to soil fractions, turnover times of bulk mineral soil showed no increase with elevation (*p*‐value = 0.2, Figure 5b). Accounting for the declining contribution of MOM fractions with increasing elevation showed that, despite longer turnover times in certain fractions at high elevations, turnover rates of the combined SOM fractions were almost the same in high and low‐elevation soils (Table 5). Similarly, bulk soil‐respired CO_2_ from a soil incubation under standardized conditions did not reveal elevational changes in their turnover times. We also computed the OC fluxes entering each pool under steady state, by dividing the OC stocks by the turnover time. The modeled OC fluxes generally did not show an elevational trend (*p* > 0.05, Table S1). The fHF was the exception, where OC fluxes decreased with increasing elevation (*p*‐value = 0.01, Table S1 and Figure S7).

**TABLE 5:** The contribution and turnover times of SOM fractions in forest soils < 500 m a.s.l. and > 1200 m a.s.l. These values were used to estimate turnover rates of POM, MOM, the mineral soil, and total SOM including mineral soil and organic layer by accounting for the relative contributions of the individual SOM fractions. Mean fractions, median turnover times, and standard errors. For POM, MOM, and total SOM, standard errors were estimated by error propagation.

	Contribution of fractions		Turnover times (years)	
	< 500 m	> 1200 m	< 500 m	> 1200 m
F‐layer	0.054 ± 0.015	0.055 ± 0.028	8.5 ± 2.4	17.5 ± 4.9
H‐layer	0.034 ± 0.113	0.168 ± 0.049	12 ± 2.0	22 ± 6.6
fLF	0.154 ± 0.060	0.164 ± 0.060	12 ± 85	13 ± 106
oLF	0.165 ± 0.023	0.198 ± 0.023	117 ± 13	156 ± 119
cHF	0.076 ± 0.014	0.086 ± 0.019	554 ± 285	915 ± 207
H_2_O_2_ oxidized fHF	0.420 ± 0.031	0.278 ± 0.019	114 ± 35	184 ± 120
H_2_O_2_ residual fHF	0.097 ± 0.039	0.047 ± 0.041	875 ± 342	1142 ± 375
POM (F + H + fLF + oLF)	0.407 ± 0.12	0.589 ± 0.086	54 ± 14	64 ± 30
MOM (cHF + fHF)	0.593 ± 0.052	0.411 ± 0.048	236 ± 55	373 ± 65
Total SOM (POM + MOM)			197 ± 57	222 ± 71

## 4 Discussion

The soils investigated in this study spanning an elevation gradient with a broad range of climatic conditions and a tree species shift from sub‐Mediterranean pubescent oak to mountain pine growing at treeline encompass the range of forest ecosystems found across Europe. Here, we observed longer ^14^C‐based turnover times and increased stocks of POM in high‐elevation forests, reflecting a reduced transformation of carbon both in the organic layer and from POM to MOM under colder conditions. However, concomitantly, the stocks of MOM decreased, with the net result being similar total SOC stocks and turnover times of bulk SOM at all elevations. This suggests that slower processing of POM and MOM in high‐elevation forests is counterbalanced by a shift in SOM composition, with a greater contribution of POM containing inherently younger SOM.

### 4.1 Increasing Turnover Times and Contributions of POM With Elevation

The increase of ^14^C‐based turnover times of the organic layer, free POM, and the mineral‐associated fHF fraction with elevation aligns with the concept that low temperatures suppress SOM processing by microbial and faunal communities. Our results agree with other ^14^C‐based case studies. For example, Leifeld et al. (2009) observed slower POM turnover rates with increasing elevation across four soil profiles on a grassland slope. Similarly, along a latitudinal gradient spanning five sites in Swedish spruce forests, Fröberg et al. (2011) reported longer turnover times of the organic layer in colder climates, attributed to lower temperatures and reduced carbon inputs from aboveground litterfall into the organic layer.

Contrary to the expectation that MOM is primarily controlled by mineralogy (Hemingway et al. 2019; Schrumpf et al. 2021) and would thus remain unrelated to elevation, we observed increasing turnover times with increasing elevation for the fHF, particularly for its oxidizable fraction (Figure 5). Consistent with the findings of Schrumpf et al. (2021), the oxidizable fHF fraction turned over relatively rapidly with turnover times similar to those of the oLF (134 vs. 109 years), indicating continuous inputs of relatively young C inputs. The oxidizable fHF was characterized by low C/N ratios (9 on average), indicative of a dominance of microbial metabolites rather than plant‐derived C, which typically exhibits high C/N ratios. This is further supported by high O/N alky‐C contributions observed in NMR‐spectra by Schrumpf et al. (2021). We therefore interpret the slower turnover of the oxidizable fHF in high‐elevation forest soils as evidence for reduced production and decomposition of microbial metabolites under their unfavorable conditions. In contrast, the residual fHF and cHF were less strongly related to elevation, indicating that other processes—such as the interaction with reactive mineral surfaces (Schrumpf et al. 2021) or contributions from petrogenic C (Evans et al. 2025)—play a more important role.

While high‐elevation forests exhibited slower C turnover times within soil fractions, they also differed in the contributions among SOM fractions and thus in SOM composition compared to low elevation forests. In agreement with our original hypothesis, SOC stocks in the organic layer increased with elevation, a pattern that was also observed in a broader dataset of Swiss forest soils including 550 profiles (Figure S1). Additionally, the POM/MOM ratio also increased with elevation. In contrast, we found that SOC stocks in the mineral soil remained relatively constant with increasing elevation, aligning with the broader dataset of Swiss forest soils (Gosheva et al. 2017). Overall, this pattern suggests a slower turnover and increasing accumulation of little‐processed OM at the expense of MOM at the high‐elevation sites. We relate this to the combined effect of a higher proportion of coniferous trees, which shed more recalcitrant litter (Augusto et al. 2015; Vesterdal et al. 2008) and the reduced microbial activity at lower temperatures. Together, these factors slow decomposition and transformation of POM, leading to a proportionally greater accumulation of POM compared to low‐elevation sites. At the same time, the reduced OM processing by microbes also results in a slower and lower production of extracellular compounds and microbial products (Liang et al. 2017), which in turn decreases microbial entombment and SOM stabilization by the interaction with reactive mineral surfaces (Cotrufo Wallenstein et al. 2013; Gies et al. 2021). In addition to reduced microbial processing, suppressed faunal activity and, consequently, lower bioturbation likely contribute to the increased proportion of POM and higher OC stocks in the organic layer with increasing elevation. In particular, soil fauna such as earthworms may play a critical role in the incorporation of POM into the mineral soil (Desie et al. 2020; Guidi et al. 2022) and for the formation of MOM (Angst et al. 2024). Cold environments with frozen soil in winter are not favorable to earthworms (Angst et al. 2024; Singh et al. 2019). In addition, the presence of polyphenol‐rich litter from coniferous trees and from ericaceous shrubs such as *Vaccinium myrtillus* forms an acidic organic layer in higher elevation forests (Desie et al. 2020). These conditions are suboptimal for earthworms (Angst et al. 2024) and other soil biota (Prescott and Vesterdal 2021). Overall, these processes result in a slower and lower transformation of POM into MOM in high‐elevation forests. This observation is consistent with global and continental‐scale surveys reporting a dominance of POM in mineral soils of cold regions, primarily in the Arctic (García‐Palacios et al. 2024), and with other altitudinal gradient studies showing high proportions of POM at high‐elevation sites (Budge et al. 2011; Khedim et al. 2023; Leifeld et al. 2009).

### 4.2 Consistent Isotopic Enrichment With Elevation

Our results show a consistent ^13^C and ^15^N enrichment from the organic layer to POM and to MOM, reflecting an increasing transformation of OM along this trajectory. Both C and N become enriched in the heavier isotopes, as the lighter isotopes are preferentially lost during the microbial processing of OM (Lorenz et al. 2020; Robinson 2001). Together with decreasing C/N ratios, this isotopic enrichment from POM and MOM thus serves as a robust indicator of organic matter transformation (Conen et al. 2008). Our ^14^C data supports this pathway of SOM transformation with C entering the soil as free POM, which is then microbially processed to MOM. Consistent with other ^14^C studies (Fröberg et al. 2011; Schrumpf et al. 2021), our ^14^C data shows that organic layers and POM in the mineral soil have decadal turnover times while MOM turns over on centennial to millennial timescales. The low C/N ratio in MOM suggests that it primarily consists of microbial metabolites (Lavallee et al. 2020; Liang et al. 2017), whereas sorption of DOM and preservation of pyrogenic carbon, which are both characterized by high C/N ratios (Brödlin et al. 2019; López‐Martín et al. 2018), seem of minor importance in the MOM. Treatment of MOM with H_2_O_2_ yielded significantly older SOM in the remaining fraction, suggesting that MOM in topsoils consists of a rather rapidly cycling fraction and a fraction with significantly slower turnover rates, the latter potentially being closely associated with reactive mineral surfaces (Kleber et al. 2007; Schrumpf et al. 2021).

We found that the C/N ratio of soil fractions increased with elevation, which we attribute to the change of tree species, with a higher C/N ratio in the conifer compared to the broadleaf forests (Figure S8). The parallel slopes for the increase in δ^13^C, δ^15^N values, and C/N ratios with elevation among the different soil fractions are to be noticed (Figure 4 and Figure S3). They suggest that the enrichment factor between POM entering the soil and the more strongly processed MOM remains constant. Consequently, the SOM transformation state of a given fraction appears independent of elevation. However, its absolute value appears to depend on the isotopic composition of the organic matter input, which progressively changes from pubescent oak in the sub‐Mediterranean zones to mountain pine at treeline over the elevation gradient.

### 4.3 Increasing POM/MOM Ratio Counterbalances Longer Turnover Times With Elevation

**FIGURE 6:**
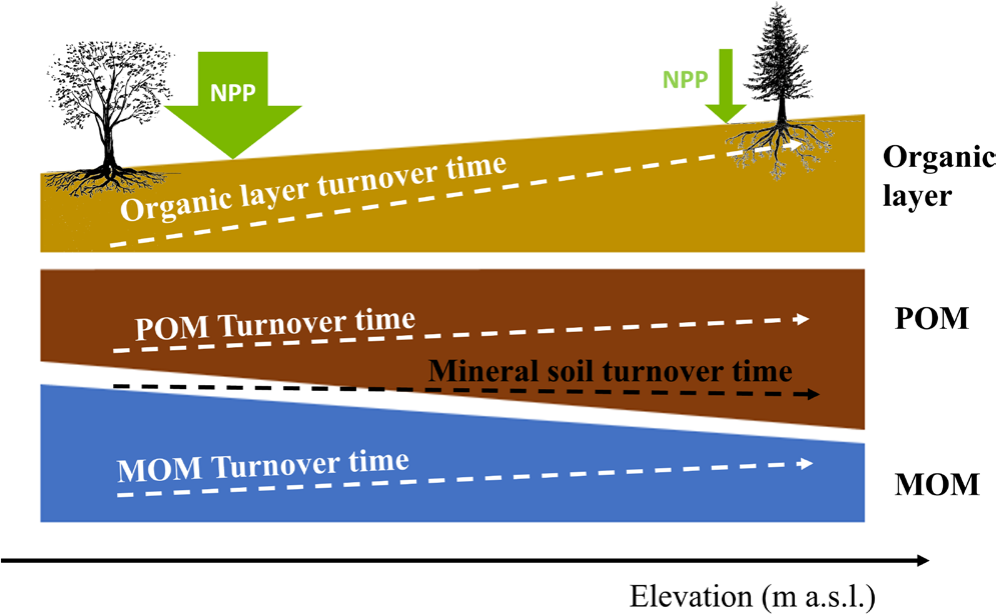
Schematic summary of findings. While SOC stocks in the organic layer (yellow) and particulate organic matter (POM) (brown) increase with elevation, they decrease in the mineral‐associated organic matter (MOM) (blue), overall shifting the POM/MOM ratio. ^14^C‐based turnover times of all individual SOC pools (indicated by dashed arrows) increase with elevations. However, turnover time of total SOC in the mineral soil remains constant as the compositional shift towards POM, which has an inherently shorter turnover time than MOM, offsets the increases of turnover times within pools.

One striking finding was that, despite the longer turnover times in the organic layer, fLF, and mineral‐associated fHF, ^14^C‐based turnover times of SOM in the bulk soil as well as in respired CO_2_ from bulk soils remained constant with elevation. In addition, the SOC stocks in the mineral soil were largely invariant along the elevation gradient—a pattern that is consistent with the larger‐scale soil survey of Swiss forest soils (Gosheva et al. 2017). We attribute the uniform turnover times of bulk SOM to a juxtaposition of changes in turnover times within soil fractions against the concomitant shift in SOM composition with elevation (Figure 6). Despite the slower turnover of POM with increasing elevation, POM remained substantially younger than MOM in low‐elevation forests (Figure 5). This inherent difference between these fractions and their fractional contribution is imprinted in the turnover of bulk SOM; in our study, the slower MOM turnover is apparently balanced out by the increased POM contribution at higher elevation. Our ^14^C‐based data are supported by litter bag studies across Swiss forests (Heim and Frey 2004) and across French forests (Fanin et al. 2020), showing smaller decomposition rates of above and belowground litter at higher elevation and with an increased presence of conifer trees. Possibly roots are the major POM source in the mineral soils, but at high‐elevation sites, the reduced microbial activity is slower so OM is also accumulating as POM which leads to small fluxes of C entering MOM through microbial entombment (Cotrufo Wallenstein et al. 2013). ^14^C‐based flux calculations—dividing pool sizes by their turnover times—indicate that as elevation increases, C fluxes through MOM decrease, which slows overall SOM turnover at least to an extent that it cancels out the higher proportion of inherently more rapidly cycling POM (Figure S6, Table S1). Combining the average changes in the distribution of individual SOM fractions across elevation with their turnover times revealed that a 70‐year increase in turnover time in the oxidized fraction over 1000 m of elevation (and a 270‐year increase in the H_2_O_2_ residual fraction) is accompanied by an 18% decline in MOM abundance over the same elevation gradient (Table 5 and Figure S2). Overall, this resulted in an almost constant turnover time of total soil organic matter including mineral soil and organic layer (+25 years or + 12%). This estimate supports the idea that the observed constant turnover times of bulk SOM with elevation are not merely an artifact of integrating different pools within the bulk soil but instead reflect concomitant changes in turnover times and relative contributions of SOM fractions. These patterns may differ in other ecosystem types (e.g., grasslands) or where ecosystem types change with elevation (e.g., transition from forest to grassland above the treeline). However, our study demonstrates that a full assessment of responses of soil C cycling to environmental changes requires consideration of both changes in SOM fractions and ^14^C‐based turnover times.

The apparent invariance in turnover times and SOC stocks in the mineral soil with elevation stands in contrast to the pronounced gradient in NPP across the Swiss forests, the latter decreasing from 0.75 kg C m^−2^ year^−1^ to 0.31 kg C m^−2^ year^−1^ with increasing elevation (Figures 1 and 6). This implies that most of the annual OM production in low‐elevation forests is decomposed within a few years (Lehmann and Kleber 2015) before it enters the mineral soil in significant amounts to imprint the bulk mineral soil ^14^C signature. Our conclusion is supported by soil respiration measurements and long‐term incubation experiments in Swiss forest ecosystems (Caprez et al. 2012; Didion et al. 2014) and is congruent with ^14^C flux modeling (Mills et al. 2013), showing that the greatest fraction of litter inputs is rapidly lost. In comparison, at high‐elevation sites, the reduced NPP balances out the slower decomposition due to lower microbial activity, rendering the SOC stocks in the mineral soil similar to those at lower elevations. Consistent with our results along our elevation gradient, Ziegler et al. (2017) observed higher decay rates and more decomposed SOM yet similar SOC stocks at warmer sites along a latitudinal gradient across the Canadian boreal forest. Collectively, these findings suggest that, despite variations in forest productivity, decomposition processes tend to result in comparable SOC stocks across different temperature regimes, while SOM composition shifts towards less transformed SOM in colder climates.

### 4.4 Implications: Vulnerability of SOC Stocks at High Elevation

Our results across our ~1700 m elevation gradient showing that SOC stocks stored in the organic layer and the contribution of POM in mineral soils increase with elevation indicate that these SOC stocks could be at risk under ongoing climate change. High‐elevation sites experience particularly strong climatic warming and hydrologic extremes (Khedim et al. 2023; Pepin et al. 2015). Given that POM is readily decomposable and temperature‐sensitive (Georgiou et al. 2024; Soong et al. 2021), these high‐elevation soils risk losing substantial amounts of C. A similar conclusion has been drawn for high latitude soils (García‐Palacios et al. 2024) and for high‐elevation grasslands (Khedim et al. 2023). Moreover, POM‐rich soils are more sensitive to the indirect effect of climate changes such as disturbances by windthrow, insect infestation, and fire, exacerbating soil OC losses (Mayer et al. 2024).

Our study also reveals constant SOM stocks along the elevation gradient from sub‐mediterranean to alpine forests with greater POM but smaller MOM stocks at higher elevation and vice versa at lower elevation. This suggests that higher temperatures promote POM processing, potentially leading to increased microbial entombment and enhanced C stabilization. Overall, our findings offer contrasting perspectives on the potential effects of warming on POM‐rich high‐elevation soils: while these soils may be more vulnerable to climate change, enhanced C stabilization at warmer temperatures through higher C transfer to stable MOM may buffer potential soil C losses by global change. Nevertheless, the risk of enhanced C losses appears increasingly likely in the face of unprecedented climatic changes and more frequent disturbances (Seidl et al. 2014; Senf and Seidl 2021).

## Author Contributions

**Margaux Moreno‐Duborgel:** conceptualization, data curation, formal analysis, investigation, methodology, visualization, writing – original draft, writing – review and editing. **Sia Gosheva‐Oney:** data curation, formal analysis, methodology, writing – review and editing. **Beatriz González‐Domínguez:** formal analysis, investigation, methodology, writing – review and editing. **Mirjam Brühlmann:** formal analysis, investigation, methodology, writing – review and editing. **Luisa I. Minich:** writing – review and editing. **Negar Haghipour:** methodology, validation, writing – review and editing. **Roman Flury:** formal analysis, investigation, validation, visualization, writing – review and editing. **Claudia Guidi:** data curation, writing – review and editing. **Alexander S. Brunmayr:** methodology, software, writing – review and editing. **Samuel Abiven:** conceptualization, writing – review and editing. **Timothy I. Eglinton:** conceptualization, funding acquisition, supervision, writing – review and editing. **Frank Hagedorn:** conceptualization, data curation, funding acquisition, methodology, supervision, validation, writing – review and editing.

## Conflicts of Interest

The authors declare no conflicts of interest.

## Supporting information


Data S1.

## Data Availability

The data and code that support the findings of this study are openly available in Zenodo at https://doi.org/10.5281/zenodo.15685173.
