# Supplementary material for: Shifting Carbon Fractions in Forest Soils Offset 14C‐Based Turnover Times Along a 1700 m Elevation Gradient
Source: Glob Chang Biol. 2025 Jul 12;31(7):e70326. doi: 10.1111/gcb.70326 (PMC12254928; doi:10.1111/gcb.70326)
Supplement: Supplementary file 1 — Data S1. [file GCB-31-e70326-s001.pdf]

Supporting information

Shifting carbon fractions in forest soils offset ^{14}C-based turnover times along a 1700 m elevation gradient

Running title: **Elevational control on ^{14}C-based SOM turnover**

Margaux MORENO-DUBORGEL[1,2], Sia GOSHEVA-ONEY[1,3], Beatriz GONZÁLEZ-DOMÍNGUEZ[3,4], Mirjam BRÜHLMANN[1,4], Luisa I. MINICH[1,2], Negar HAGHIPOUR[2,5], Roman FLURY[1], Claudia GUIDI[1], Alexander S. BRUNMAYR[2,6], Samuel ABIVEN[7,8], Timothy I. EGLINTON[2], Frank HAGEDORN[1]

[1]Swiss Federal Institute for Forest, Snow and Landscape Research (WSL), Switzerland
[2]Department of Earth and Planetary Sciences, ETH Zurich, Switzerland
[3]Department of evolutionary Biology and environmental Studies, University of Zurich (UZH), Zurich, Switzerland
[4]Department of Geography, Soil Science and Biogeochemistry Unit, University of Zurich (UZH), Zurich, Switzerland
[5]Laboratory for Ion Beam Physics, Department of Physics, ETH Zurich, Switzerland
[6]Department of Physics, Imperial College London, United Kingdom
[7]Laboratoire de Géologie, CNRS - École normale supérieure, PSL University, Paris, France
[8]Centre de Recherche en Ecologie Expérimentale Et Prédictive (CEREEP-Ecotron Ile de France), Ecole Normale Supérieure, CNRS, PSL Research University, Paris, France

Correspondence to: M. Moreno-Duborgel (margaux.duborgel@wsl.ch)

POM accumulation at extreme moist and dry sites

In our study, 4 out of the initial 54 sites were identified as “outliers” that do not follow the elevational patterns. These soils have particularly high SOC stocks in the organic layer or high POC contents compared to other sites at the same elevation and experience peculiar conditions independent from elevation. At one extreme, some sites are waterlogged, where SOM decomposition is reduced due to anaerobic conditions hindering microbial activity (Wang et al., 2018). A larger scale assessment of Swiss forest soils based on 1000 profiles, Gosheva (2017) revealed about 10% waterlogged sites with atypical SOC stocks. At the other extreme, soils experience very dry conditions, at least temporarily reducing SOM transformation. These soils are characterized by Xeromoder-type organic layer and particularly high stone content, which may hydrologically disconnect the organic layer and the topsoil from the underlying mineral soils and further reinforce the drying effect.

Due to its leverage on models, we also removed ^{14}C-based SOM turnover rates of a treeline site at 2020 m a.s.l. (Stillberg) from the data analysis. Nonetheless, this site is in agreement with the elevation pattern. We regard the estimated turnover times of 168 years for the H-layer and even 1182 years the fLF in the mineral soil under the 10 to 20 cm thick organic layer as an indication that under the harsh climatic conditions at treeline and litter inputs deriving from polyphenol-rich dwarf shrubs (Hagedorn et al., 2010), transformation of litter and POM can be extremely slow.

Initial conditions for ^{14}C-based turnover time modeling

The function OnepModel14() from the *SoilR* package (version 1.2.107) (Sierra et al., 2014) that we used to model turnover times, requires the initial conditions of the pool at the time when the simulation starts (i.e., in our case, in year 1850). As we considered that the pools are at steady state conditions, we assumed that the initial C stock is the actual C stock in kg C m^{-2}. Under the steady state condition, the input of C into the pool equals k * *C stock*. The initial F^{14}C of the pool is equal to $\frac{k}{k+\lambda}$.

Under steady state conditions, $\frac{d(F)}{dt} = 0$ and $C_i = C_{i+1} = \frac{I}{k}$

And we assume that before 1950 $F^{14}C_{atm} = 1$ (Torn et al., 2009)

$$\frac{d(F*C_i)}{dt} = I * F_{atm,i} - (k+\lambda)(F_i * C_i)$$

$$0 = \frac{I}{C_i} - (k+\lambda) * F_i$$

$$F_i = \frac{k}{k+\lambda}$$

Supplementary figures and tables

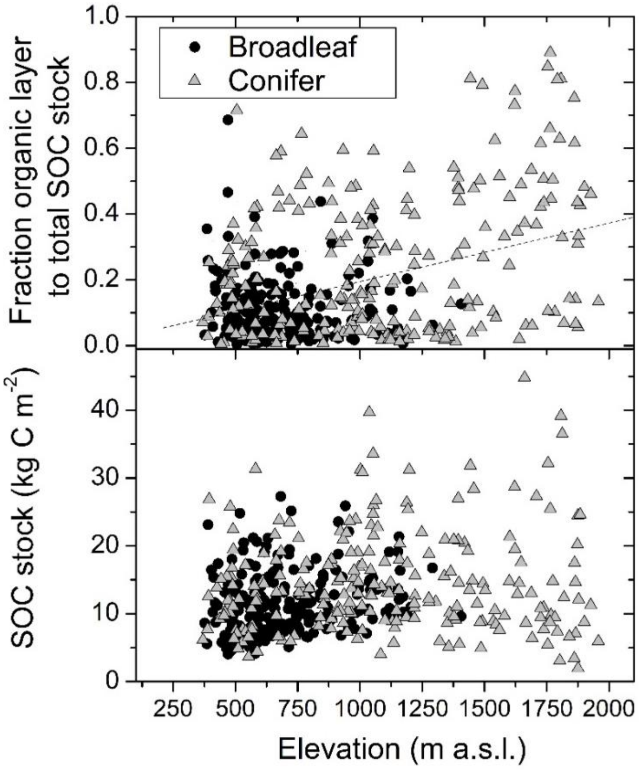

Supplementary Fig. 1: Elevation pattern of the fraction of C stock in the organic layer on the total SOC stock down to bedrock in forest sites across Switzerland (*p*< 0.001, R^2 = 0.16) (top graph). b) SOC stocks down to the bedrock. (n = 556; data from Gosheva et al., 2017) (bottom graph).

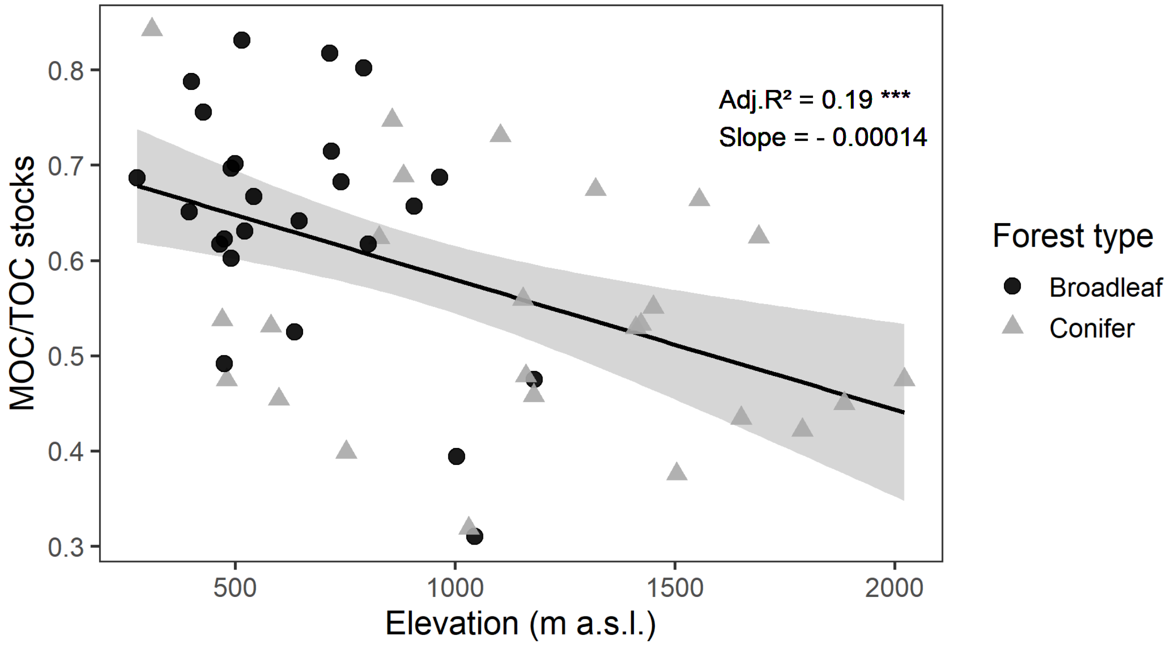

Supplementary Fig. 2: Elevation trend of the MOC/TOC stock ratio.

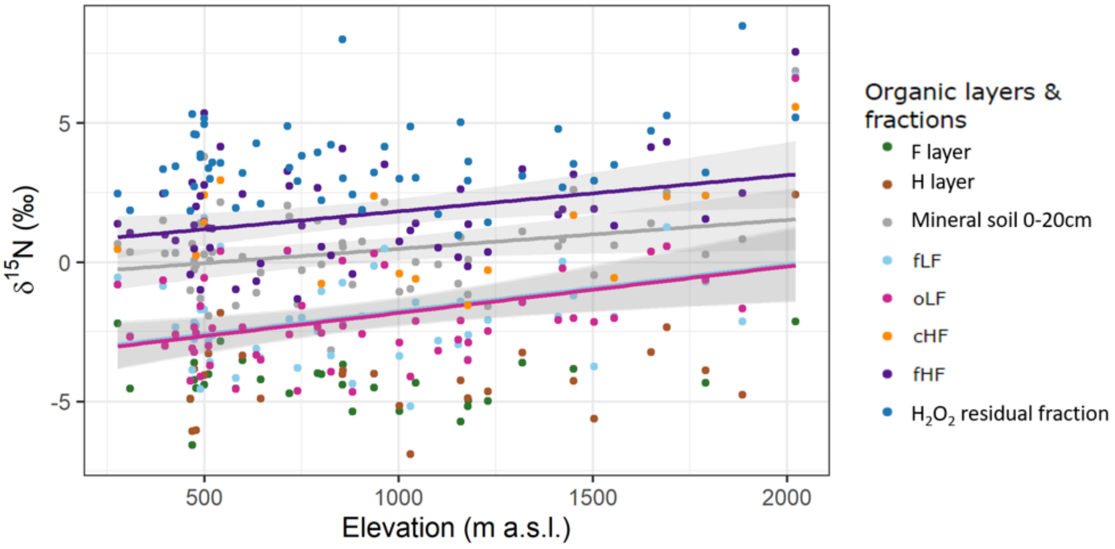

Supplementary Fig. 3: Linear relations between elevation and $\delta^{15}N$ (‰) in SOC fractions. Only significant relations are shown.

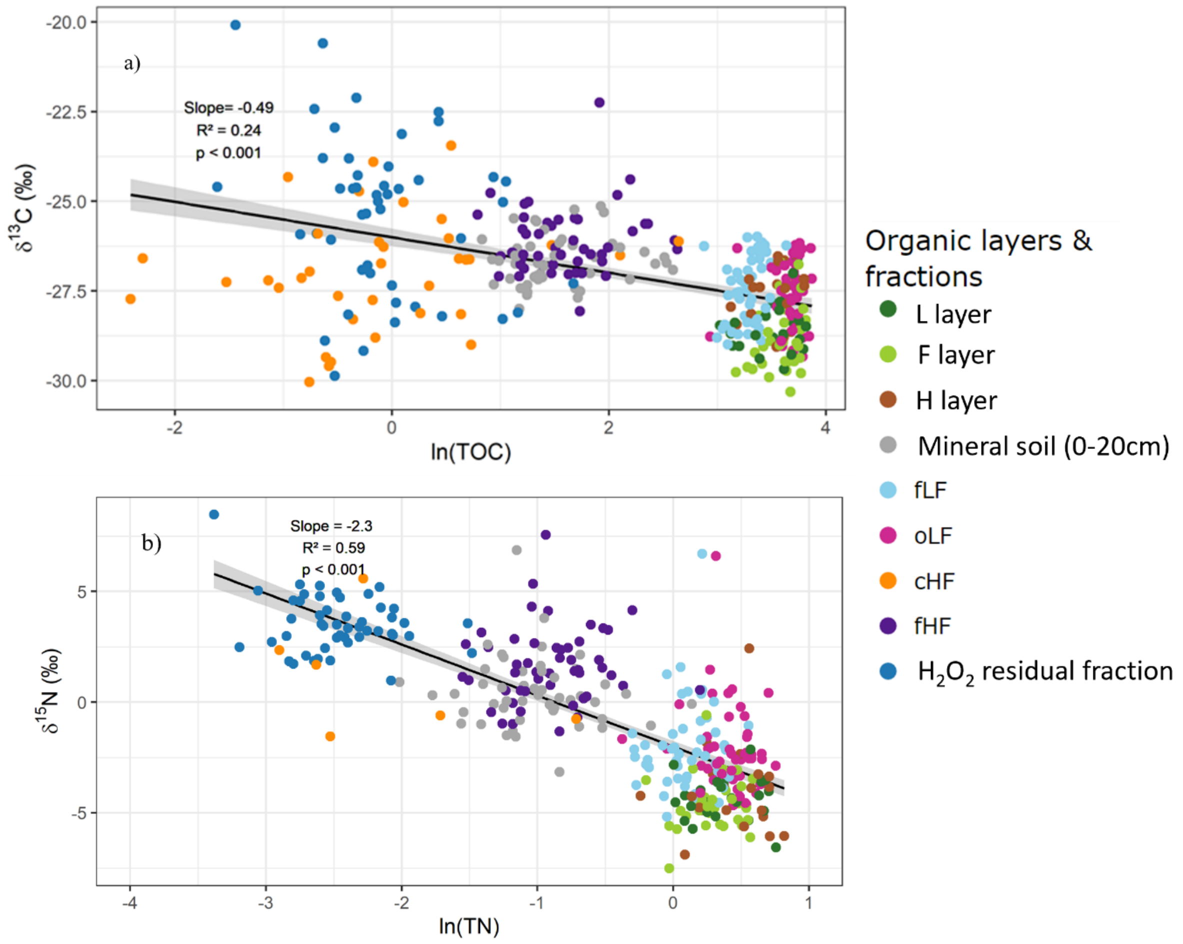

Supplementary Fig. 4: a) Logarithmic carbon concentration against $\delta^{13}C$ in the organic layers and mineral soil fractions. There is an enrichment in $\delta^{13}C$ from organic layers to mineral-associated organic matter fractions the slope of the linear regression is the $\delta^{13}C$ enrichment factor from the organic layer to the MOM. b) Logarithmic nitrogen concentration against $\delta^{15}N$ in the organic layers and mineral soil fractions. The linear regression shows an enrichment in $\delta^{15}N$ from organic layers to mineral-associated organic matter fractions.

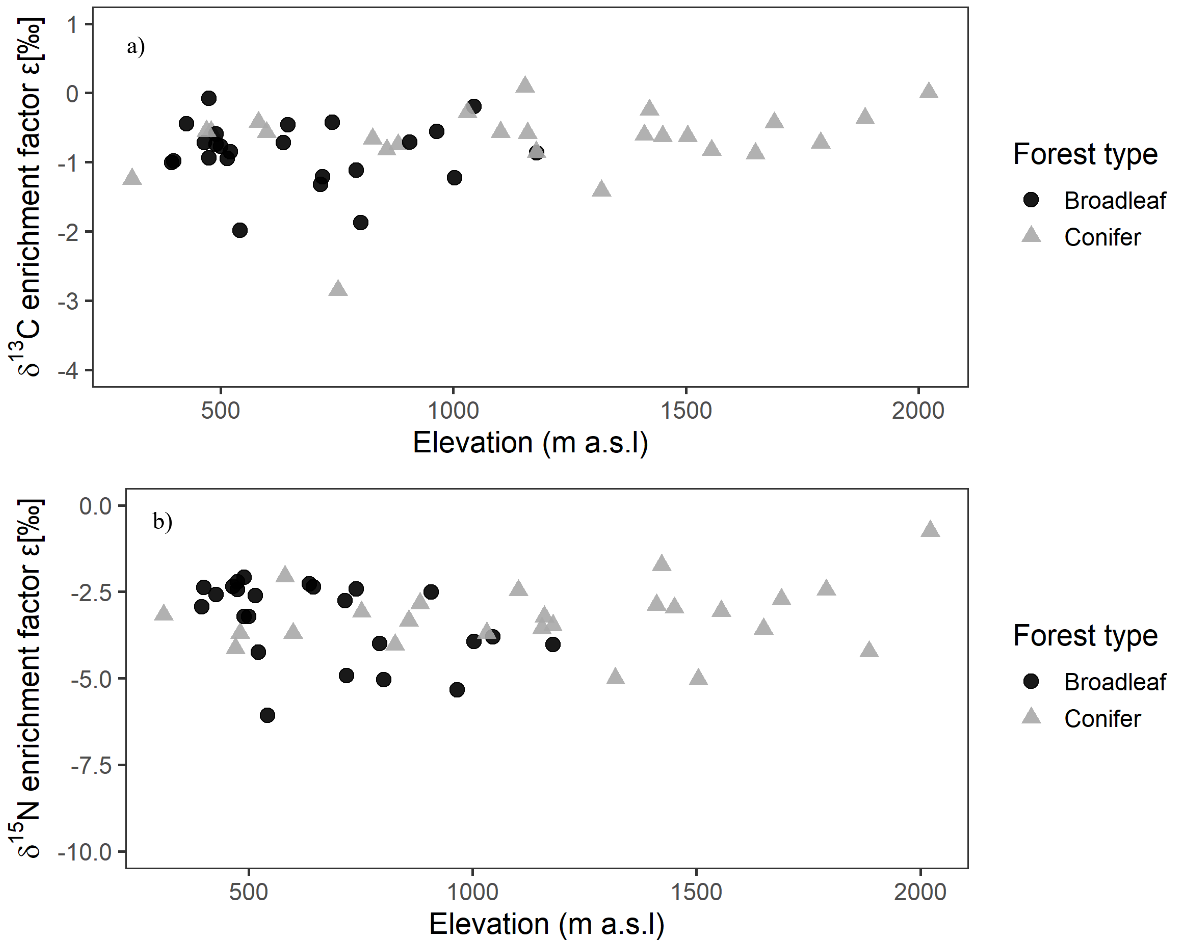

Supplementary Fig. 5: No elevation trend of a) δ^{13}C enrichment and b) δ^{15}N enrichment factors. The elevation effect was tested with linear models ($p_{13C\ enrichment} = 0.08$, $p_{15N\ enrichment} = 0.9$)

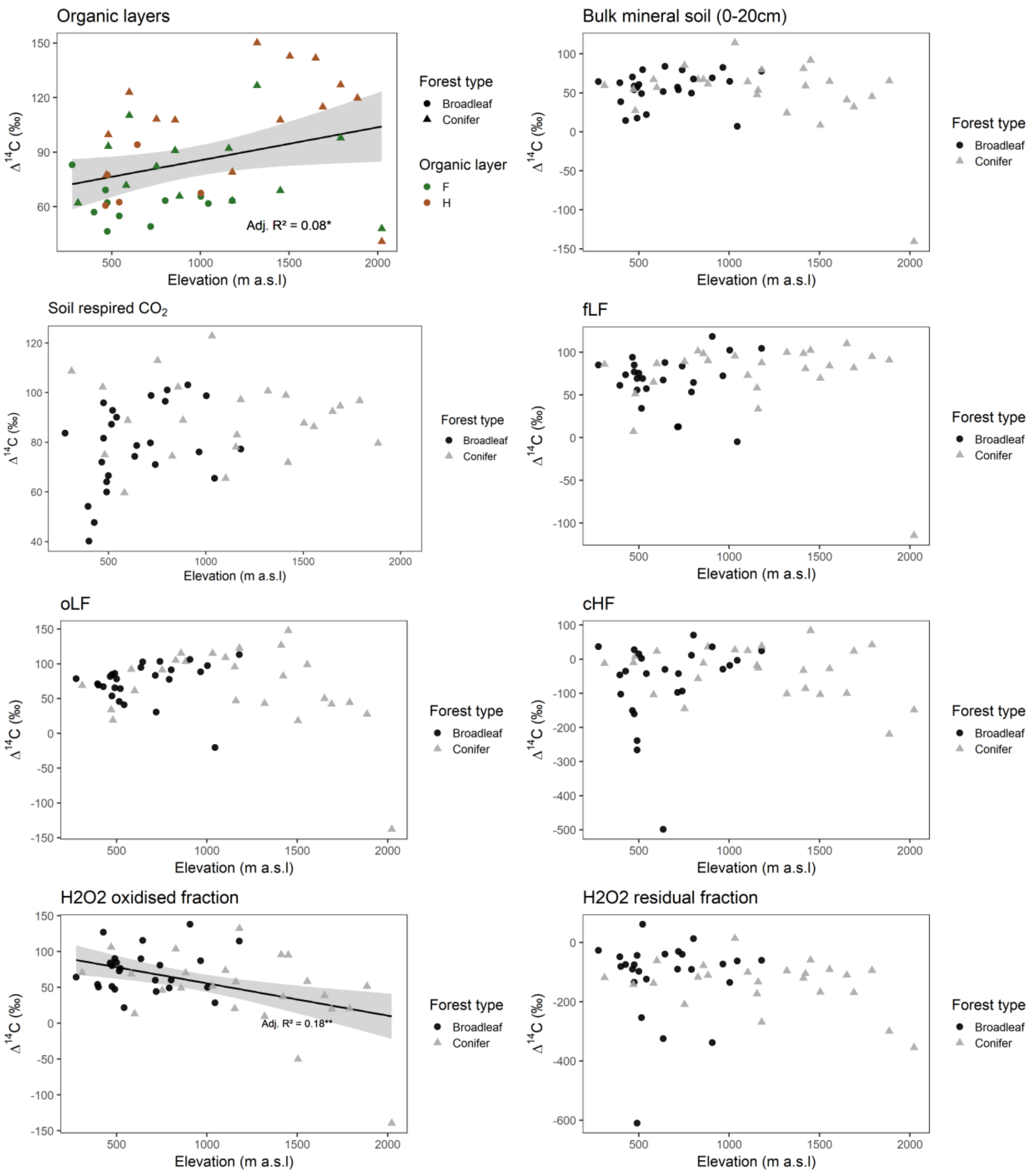

Supplementary Fig. 6: Elevation influence on the $\Delta^{14}C$ (‰) content in the organic layers, bulk soil (0-20cm) and in soil fractions. Only significant relations with elevation are shown.

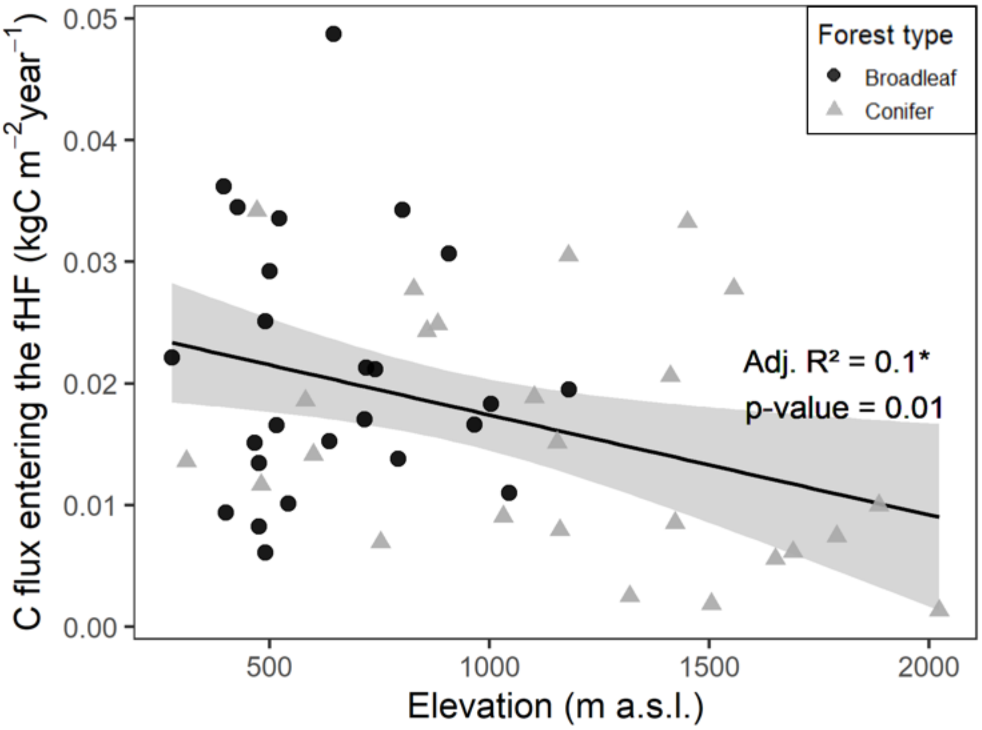

Supplementary Fig.7: Modeled OC flux entering the fine heavy fraction (fHF) in kgC m^{-2} $year^{-1}$. The OC flux was obtained under the steady state assumption by dividing the SOC stock in the fHF by the ^{14}C-derived turnover time.

Supplementary Table 1: p-values of linear models testing if there is linear relation of the OC fluxes along elevation in the different SOC fractions. The fluxes were obtained, assuming a steady state, by dividing the SOC stocks by the ^{14}C-modeled turnover time.

**Fluxes (kgC m^{-2} $year^{-1}$)**		**L layer**	**F layer**	**H layer**	**Bulk mineral soil (0-20cm)**	**fLF**	**oLF**	**cHF**	**fHF**	**oxidised**	**residual**
**Elevation (m a.s.l.)**	**p-value**	**N.A**	0.08 n.s	0.7 n.s	0.8 n.s	0.7 n.s	0.2 n.s	0.5 n.s	**0.01***	0.09 n.s	0.08 n.s

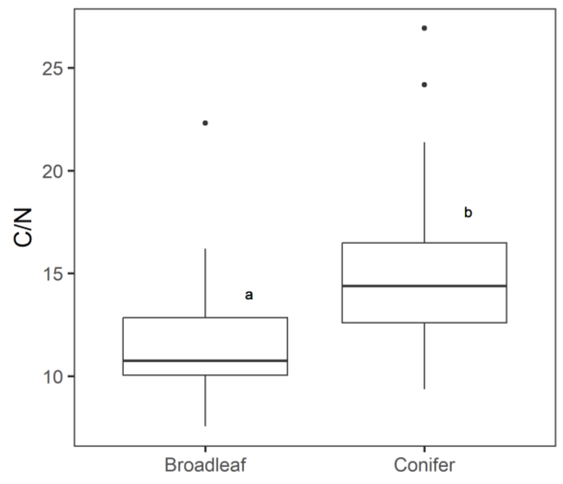

Supplementary Fig. 8: C/N ratio in broadleaf and conifer forest bulk mineral soil (0-20cm). The C/N ratio in the soil from the two different forests are significantly different ($p < 0.001$; Wilcox).

We conducted a post hoc variance partitioning analysis based on the models in Table 2. The results indicate that the SOM fraction explains most of the variance across all response variables (Supplementary Fig.9). This fact supports our approach of investigating each SOM fraction individually with separate models (see Table 4).

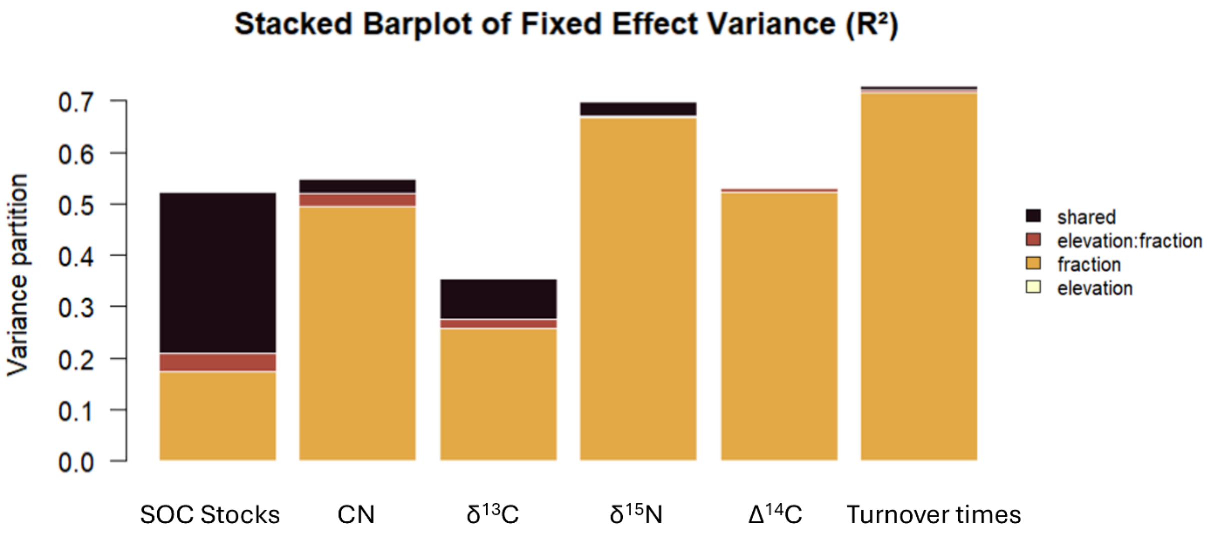

Supplementary Fig.9: Variance partitioning analysis.

References

Gosheva, S. G. (2017). The Drivers of SOC Storage: The Effect of Climate, Forest Age, and Physicochemical Soil Properties in Swiss Forest Soils.

Hagedorn, F., Martin, M., Rixen, C., Rusch, S., Bebi, P., Zürcher, A., Siegwolf, R. T. W., Wipf, S., Escape, C., Roy, J., & Hättenschwiler, S. (2010). Short-term responses of ecosystem carbon fluxes to experimental soil warming at the Swiss alpine treeline. Biogeochemistry, 97(1), 7–19. https://doi.org/10.1007/s10533-009-9297-9

Sierra, C. A., Müller, M., & Trumbore, S. E. (2014). Modeling radiocarbon dynamics in soils: SoilR version 1.1. Geoscientific Model Development, 7(5), 1919–1931. https://doi.org/10.5194/gmd-7-1919-2014

Torn, M. S., Swanston, C. W., Castanha, C., & Trumbore, S. E. (2009). STORAGE AND TURNOVER OF ORGANIC MATTER IN SOIL. In Soil and Water Chemistry (pp. 176–227). https://doi.org/10.1201/b18385-8

Wang, C., Houlton, B. Z., Liu, D., Hou, J., Cheng, W., & Bai, E. (2018). Stable isotopic constraints on global soil organic carbon turnover. Biogeosciences, 15(4), 987–995. https://doi.org/10.5194/bg-15-987-2018
